# A prospective cohort study of the incidence, etiology and outcome of pneumonia among cancer patients in an oncology intensive care unit from Eastern India

**DOI:** 10.3332/ecancer.2025.1970

**Published:** 2025-08-19

**Authors:** Simran Malik, Sudipta Mukherjee, Pralay Shankar Ghosh, Santanu Bagchi, Gaurav Goel, Soumyadip Chatterji, Saugata Sen, Debashree Guha Adhya, Sangeeta Das Bhattacharya, Sanjay Bhattacharya

**Affiliations:** 1 School of Medical Science and Technology, Indian Institute of Technology, Kharagpur, West Bengal, India; 2 Department of Microbiology, Tata Medical Center, Kolkata, West Bengal, India; 3 Department of Critical Care, Tata Medical Center, Kolkata, West Bengal, India; 4 Department of Infectious Diseases, Tata Medical Center, Kolkata, West Bengal, India; 5 Department of Radiology, Tata Medical Center, Kolkata, West Bengal, India; 6 Section of Internal Medicine and Pediatrics, Christiana Care Health Systems, Newark, DE, USA

**Keywords:** pneumonia, cancer, oncology ICU, clinical outcome, risk factors, LMIC

## Abstract

With the rising incidence of cancer in low and lower-middle-income countries, the burden of pneumonia, which causes disproportionate morbidity and mortality in cancer patients, presents an evidence gap.

We conducted a 1-year prospective cohort study in the oncology ICU at Tata Medical Center Kolkata, to determine the incidence, risk factors, etiology and impact of pneumonia on length of stay and mortality. Pneumonia was identified via daily ICU rounds using clinical and radiological features. A 1:1 age and sex matched comparison cohort without pneumonia was included. Etiology of pneumonia was determined using microscopy, culture, ELISA, PCR and cartridge-based nucleic acid amplification tests. Logistic regression was used to study risk factors, Cox regression for mortality and linear regression for hospital and ICU length of stay.

There were 2279 ICU admissions. We recruited 711 patients: 355 had pneumonia, 356 did not. The incidence of pneumonia was 15.6% (95% CI: 14.1%–17.1%); 51.8% were community-acquired (CAP) and 48.2% were hospital-acquired (HAP). Seventy percent of CAP patients experienced recent healthcare exposure, with 28% hospitalised.

All-cause 90-day mortality (6.4 per 1000 person-days) was 9 times higher in pneumonia patients. Lengths of hospital stay (18.6 days), ICU stay (10.9 days) and mechanical ventilation (2 days) were higher in pneumonia patients.

Seventy-three percent of pneumonia patients had positive microbiology from lower respiratory samples. Gram-negative bacilli were frequent in both CAP and HAP. Influenza A/B was frequent in the monsoon and *Haemophilus influenzae* in the winter.

Bone-marrow transplant, hematological malignancies, neutropenia and chronic obstructive pulmonary disease increased pneumonia risk. CAP, hematological malignancies and neutropenia increased the risk of death by day 90.

Pneumonia, both CAP and HAP, increased mortality and hospital and ICU length of stay in adults with cancer. Gram-negative bacilli were common in both CAP and HAP. Tailored infection control programmes and an emphasis on adult vaccination are imperative to pneumonia prevention.

## Introduction

Globally, nearly 24 million people were diagnosed with cancer in 2019, and 1.2 million in India. [1, 2] The incidence of cancer is rising, with the greatest burden in lower (LICs) and lower middle-income countries (LMICs). [3] Implementation research is required from LICs/LMICs to tackle the challenges of providing cancer care [4].

Thirty-two million cases of pneumonia occurred globally in 2021, and 10.1 million in India. [5]. Cancer-specific burden of pneumonia is not adequately known, especially in south east Asia. Pneumonia can cause disproportionate morbidity and mortality in cancer patients. [6] Hospitalisation from pneumonia in cancer patients may result in increased length of stay, complexity and cost [7].

Immune dysfunction due to disease and treatment and repeated encounters with health systems increase the risk of pneumonia from resistant bacterial pathogens, *Mycobacterium tuberculosis*, fungi and viruses, including SARS-CoV2 [8]. Multidrug-resistant species such as methicillin-resistant *Staphylococcus aureus*, cephalosporin-resistant gram-negatives and intrinsically antibiotic-resistant species like *Stenotrophomonas*, *Burkholderia* are frequent [9].

Hospital-acquired pneumonia (HAP), including ventilator-associated (VAP) and non-ventilator-associated HAP (NV-HAP), are important quality indicators for hospital infection control programs [10]. They cause prolonged hospitalisation and worse clinical outcomes [11]. The NV-HAP rate in LMICs is not well documented. The VAP rate in India and LMICs is 14.3 and 13.4 per 1,000 days of ventilator use, respectively [12]. These are higher than VAP rates in upper-middle income countries (11.8) and high-income countries (8.8) [12].

The burden of pneumonia in the oncology ICU, risk factors and etiology in India presents an evidence gap requiring systematic surveillance. We conducted a prospective cohort study over 1 year to determine the incidence, risk factors, etiology and impact of pneumonia in terms of hospital length of stay and mortality in adults admitted to the oncology ICU in eastern India.

## Methods

### Study setting and design

This study was conducted in the adult ICU of Tata Medical Center Kolkata (TMCK), a 437-bed tertiary cancer center serving east and north-east India, Bangladesh and Bhutan.

All patients admitted to the ICU from October 1, 2022 to September 30, 2023 were assessed for inclusion. This was an observational study and did not interfere with patient care. It was approved by the Institutional Ethics Committee of TMCK (ID: 2022/TMC/252/1RB23) and the need for informed consent was waived.

### Selection of participants

The case identification team included ICU, infectious disease and clinical microbiology physicians, a research fellow and infection control nurses. Through daily ICU rounds, the team identified pneumonia cases based on the CDC National Health Safety Network (NHSN) criteria [13]. Clinical and radiological findings suggestive of pneumonia (infiltration/consolidation/cavitation) were essential for diagnosis (Supplemental Table S1). Microbiological confirmation was desirable. The date of the event was defined as the date of the first positive chest radiology (ultrasound (USG), chest X ray or CT scan).

A 1:1 age and gender matched comparison cohort of patients admitted to the ICU during the same time was included. These individuals had malignancy but did not have pneumonia at the time of admission to the ICU or during the course of hospitalisation.

### Definitions

Pneumonia was further classified as CAP and HAP based on the definitions by the American Thoracic Society/Infectious Diseases Society of America (ATS/IDSA) [14] and Healthcare Association Infection Surveillance India [15].

- Community-acquired pneumonia was defined as pneumonia present on admission, with clinical features and imaging (USG/chest X-ray/CT scan) within two calendar days of hospital admission.
- Hospital-acquired pneumonia was defined as pneumonia with clinical features and imaging (USG/chest X-ray/CT) after over two calendar days of hospital admission or within two calendar days of discharge from the hospital.

HAP was further sub-classified as VAP and NV-HAP.

- Ventilator-associated pneumonia was a HAP with the clinical features and imaging (USG/chest X-ray/CT) occurring after two consecutive calendar days of intubation and mechanical ventilation.
- Non-ventilator-associated HAP fulfilled the HAP criteria but occurred in the absence of or in less than two calendar days of intubation and mechanical ventilation.

### Data collection

- A standard case record form was used to collect demographic and treatment information. Physical and electronic medical records (via the Hospital Management System) were used to identify clinical features of pneumonia, dates of admission, discharge and survival at 30, 60 and 90 days.
- The pictures archiving and communication system was used to access radiology reports and physical medical records were reviewed for point of care USG evaluation of the lung.
- All patients present in the ICU during the surveillance period were included in the calculation of the denominator.
- All patients in the ICU who were on mechanical ventilators were considered for the calculation of ventilator days and VAP rate.

### Microbiology

Microbiology testing involved microscopy, culture, ELISA, PCR and cartridge-based nucleic acid amplification tests (CB-NAATs, namely Cepheid’s GeneXpert, bioMérieux’s Biofire, Qiagen’s QIASTAT) for the detection of bacteria, fungi, mycobacteria and viruses. A combination of tests was done as clinically required. Sputum, endotracheal aspirate, bronchoalveolar lavage and nose and throat swab samples for 14 days starting at the date of the event were considered. [16] Samples with *Enterococcus* were considered contaminated, and those with coagulase-negative *Staphylococci*, *Candida* and normal respiratory flora were excluded [13].

### Outcomes

The main outcome measures in this study were the incidence of pneumonia and the impact of pneumonia on hospital length of stay and mortality at 30, 60 and 90 days. Other outcome measures include analysis of risk factors, determination of etiology and seasonality.

### Statistical analysis

Data analysis was done in *STATA-17*.

- Using descriptive analysis, frequencies and proportions of categorical, demographic and comorbidity characteristics were calculated in the cohorts and compared.
- The mean lengths of hospital stay and ICU stay were compared using linear regression.
- The all-cause 30-, 60- and 90-day mortality for the two cohorts was compared using Cox regression hazard ratio. Mortality attributable to pneumonia was calculated at each time point. Kaplan–Meier survival analysis and log-rank test were performed to compare all-cause mortality by 90 days in the two cohorts.
- The frequency and proportion of each pathogen detected in the respiratory samples of patients with pneumonia were reported using the total number of cases that had a positive microbiology as the denominator. A treemap was constructed to depicted relative detection rates of pathogens. The frequency of each pathogen in CAP and HAP was compared using the Z-test.
- We considered four seasons: winter (January–March), pre-monsoon/summer (April–June), monsoon (July–September) and post-monsoon (October–December) as defined by the Indian Meteorological Department [17]. The proportion of pneumonia cases where each pathogen was detected across the seasons was compared using chi-square tests for homogeneity.
- Logistic regression was performed to determine the risk factors for pneumonia.
- Cox regression was performed to determine the predictors of all-cause mortality in patients with pneumonia.
- Linear regression was performed to identify the determinants of length of hospital stay and length of ICU stay in patients with pneumonia.
- Multivariable models for logistic regression, Cox regression and linear regression included all variables having *p*-value ≤ 0.10 in univariate analysis. The penalised maximum likelihood approach proposed by Firth [18] was used for multivariable logistic regression for risk factors of pneumonia.
- Days of mechanical ventilation and respiratory assist device use were compared using two-sample tests of proportions. The mean number of days of mechanical ventilator use and respiratory assist device use were compared for patients with pneumonia and those without, using the Z-test.

## Results

### Patient characteristics

There were 2,279 admissions to the adult ICU from October 1, 2022 to September 30, 2023. We recruited 711 patients: 355 had pneumonia and 356 did not. Age, sex and BMI of the patients in the two cohorts were comparable. Hematologic malignancies were more common in patients with pneumonia and solid organ tumours were more common in patients without (Table 1).

### Incidence of pneumonia

Three hundred fifty-five episodes of pneumonia occurred over 1 year, with an incidence of 15.6% (95% confidence interval (95% CI) =14.1–17.1%). Twenty-five cases (7.0%) were identified as recurrence of pneumonia. Monthly incidence ranged from 19.5% in October 2022 to 9.1% in April 2023.

Sixty percent of patients with pneumonia had new onset or worsening cough or dyspnea or tachypnea, 40.9% had worsening gas exchange and 39.5% had fever. Radiological features were present in all patients with pneumonia: consolidation (81.1%) was the commonest, followed by infiltration (19.7%) and cavitation (2.3%) (Supplemental Table S2).

The incidence of CAP was 51.8% (184/355) and HAP was 48.2% (171/355). Twenty-six cases (15.2% of HAP) were VAP and 145 (84.8%) were NV-HAP. Nine out of 26 VAP cases (34.6%) were post-surgical. Over the study period, 2,364 ventilator days were recorded, with a VAP rate of 11.0 per 1000 ventilator days. January 2023 had the lowest VAP rate (0 per 1000 ventilator days), whereas July 2023 had the highest (29.3 per 1,000 days of ventilator days).

### Clinical outcomes

**Figure 1.**
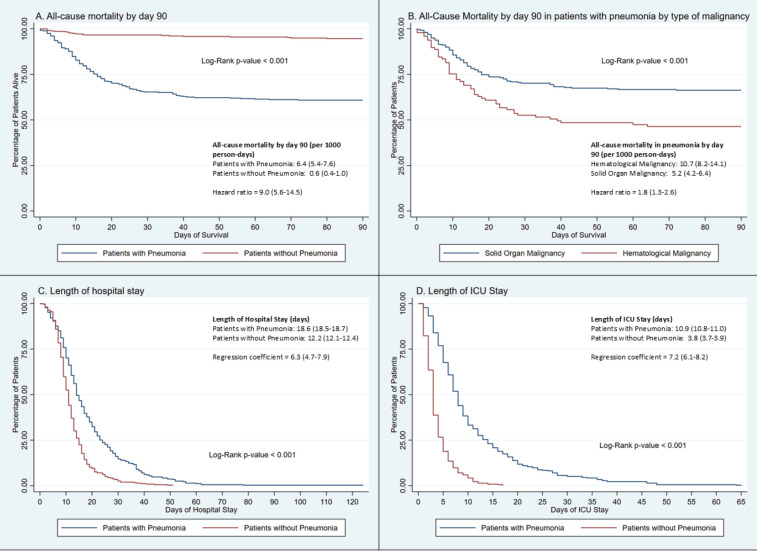
Kaplan-meier plots for clinical outcomes in patients with pneumonia and patients without pneumonia.

Patients with pneumonia had higher all-cause mortality at days 30, 60 and 90 (*p* < 0.001). Pneumonia patients had a 9 times increased risk (hazard ratio = 9.0, 95% CI: 5.6–14.5) of mortality at day 90 (Figure 1). The all-cause mortality rate in pneumonia at day 30 was 14.6 deaths per 1,000 person-days, 9.1 at day 60 and 6.4 at day 90. Of these 13.1 (95% CI: 10.8–16.1), 8.2 (95% CI: 6.1–9.7) and 5.8 (95% CI: 4.7–6.9) deaths per 1,000 person-days, respectively, were attributable to pneumonia. The majority of deaths occurred in the first 30 days (Table 2, Figure 1). The all-cause mortality in patients with hematological malignancy (10.5 deaths per 1,000 person-days) was two-fold higher than in patients with solid organ malignancy (5.2 deaths per 1,000 person-days). All mortality occurred in the first five weeks from the date of the event in both sub-groups.

The mean length of hospital stay in patients with pneumonia was 18.6 days (95% CI: 18.5–18.7), which was significantly higher (*p* < 0.001) than in patients without pneumonia (12.2 days, 95% CI: 12.1–12.4). The mean length of ICU stay in patients with pneumonia was 10.9 days (95% CI: 10.8–11.0) compared to 3.8 days (95% CI: 3.7–3.9) in patients without pneumonia (Table 2). Two-hundred ninety-five of 355 patients with pneumonia (83.1%, 95% CI: 79.2%–87.0%) required mechanical ventilation compared to 95 of 356 patients without pneumonia (26.7%, 95% CI: 22.1%–31.3%, *p* < 0.001). The mean ventilator days in pneumonia was 2.0 (95% CI: 1.9–2.1) compared to 0.4 (95% CI:0.3–0.5, *p* < 0.001) in the non-pneumonia group. Respiratory assist device was used in 47.6% (95% CI: 42.4%–52.8%) of patients with pneumonia compared to 26.7% (95% CI: 22.1%–31.3%) of patients without pneumonia. The mean duration of respiratory assist device use was 10 times higher in patients with pneumonia (8 days, 95% CI: 7.9–8.1).

- Patients with VAP and CAP had higher all-cause mortality at days 30, 60 and 90 than patients with NV-HAP (*p* < 0.05).
  - At day 30, the mortality rate was 18.7 deaths per 1,000 person-days (95% CI: 10.6–33.0) in VAP, 10.1 (95% CI: 7.3–13.9) in NV-HAP and 18.1 (95% CI: 14.4–22.7) in CAP.
  - At day 60, the mortality rate was 11.3 (95% CI: 6.4–19.9) in VAP, 6.2 (95% CI: 4.6–8.4) in NV-HAP and 11.4 (9.2–14.2) in CAP.
  - At day 90, the mortality rate was 8.1 (95% CI: 4.6–14.3) in VAP, 4.4 (95% CI: 3.2–5.9) in NV-HAP and 8.2 (95% CI: 6.6–10.1) in CAP.
- The mean length of hospital stay in VAP was 28.9 days (95% CI: 23.5–34.4), which was significantly higher than NV-HAP (22.4 days, 95% CI: 19.9–24.9) and CAP (14.1 days, 95% CI: 12.6–15.6) (*p* < 0.05).
- The mean length of ICU stay was also significantly higher in patients with VAP (21.3 days, 95% CI: 15.8–26.7) compared to NV-HAP (11.6 days, 95% CI: 9.9–13.3) and CAP (9.0 days, 95% CI: 7.9–10.1).
- While there was no significant difference in the use of mechanical ventilation or respiratory assist devices, we observed increased days of ventilator use in HAP (9.0 days, 95% CI: 8.9–9.2) compared to CAP (7.0 days, 95% CI: 6.9–7.2).

### Microbial etiology of pneumonia

Two-hundred and fifty-eight cases (72.7%) had positive microbiology: 75.0% CAP, 70.2% HAP. Two or more pathogens were detected in 47.7% of cases and these were classified as co-infections. The incidence of bacterial pneumonia was 29.1%, viral pneumonia 14.3%, fungal pneumonia 7.4% and mycobacterial pneumonia 1.6%. Bacterial pneumonia was more common in HAP (*p* = 0.006). Co-infections were more common in CAP (*p* = 0.04).

**Figure 2.**
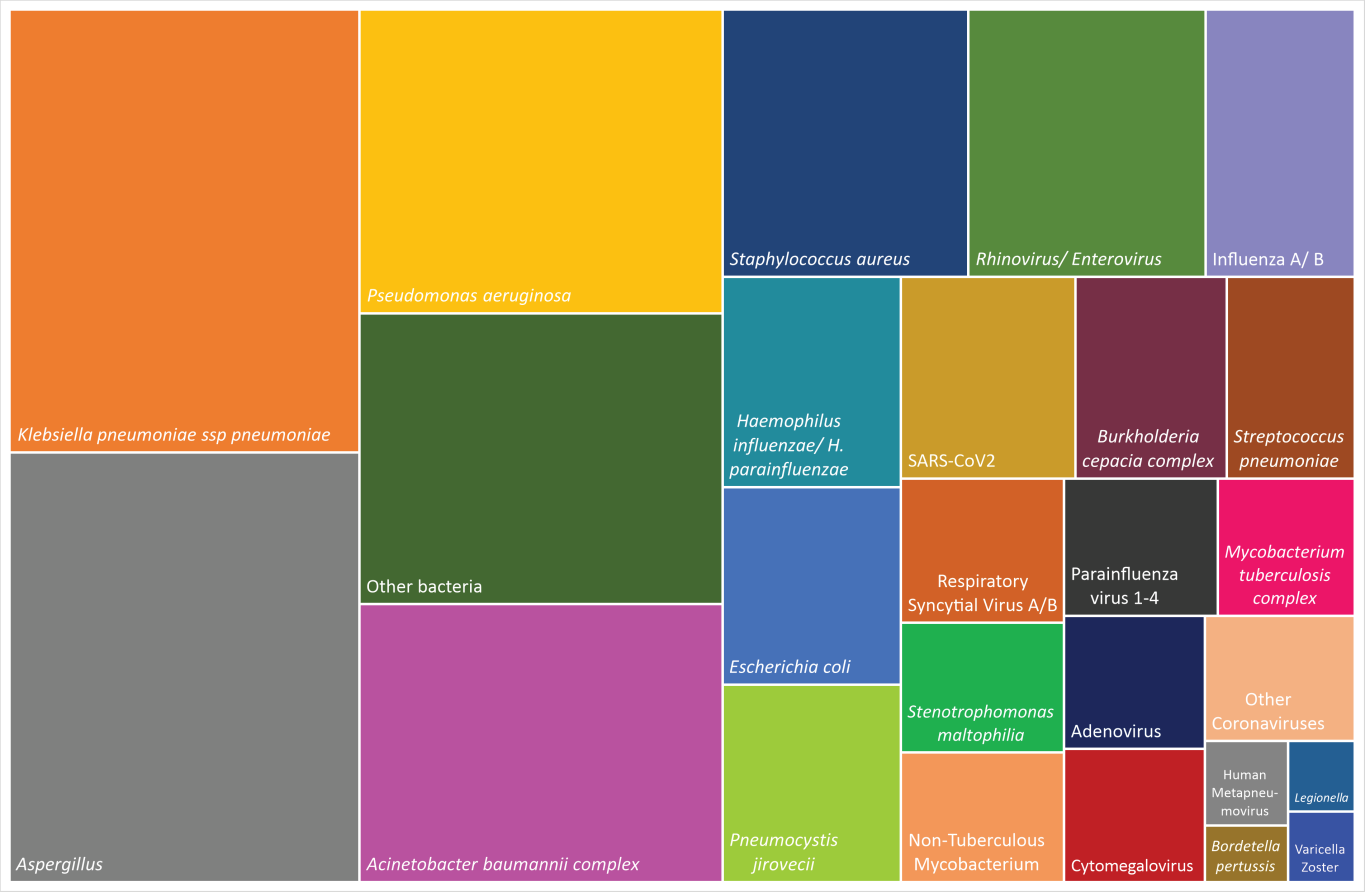
Treemap for the prevalence of each pathogen in cases of pneumonia with a microbiological confirmation (*n* = 258). (a): Other bacterial species include *Streptococcus agalactiae, Elizabethkingia meningoseptica, Aeromonas spp., Proteus spp., Enterobacter cloacae, Serratia marcescens, Moraxella catarrhalis, Acinetobacter lwofii, Klebsiella aerogenes, K. oxytoca, Escherichia fergusonii, Achromobacter denitrificans, Nocardia, Chryseobacterium indologenes, Morganella morganii, Cuprividus pauculus*. (b): Non-tuberculous *Mycobacterium include Mycobacterium abscessus, M. paraense, M. simiae, M. lentiflavum*.

Gram-negative bacteria were the most prevalent pathogens, including *Klebsiella pneumoniae* (66 cases)*, Pseudomonas aeruginosa* (47) and *Acinetobacter baumannii* complex (43). *Staphylococcus aureus* (28) was frequent as well. *Haemophilus influenzae* was detected in 15 cases, *Streptococcus pneumoniae* in 11 and *M. tuberculosis* in 8. *Pneumocystis jirovecii* was detected in 15 cases. Rhinovirus/enterovirus were the most common viruses (27), followed by influenza A/B (17), SARS-CoV2 (15) and RSV A/B (10) (Figure 2).

*Staphylococcus aureus*, *S. pneumoniae* and RSV A/B were all more common in CAP (*p* < 0.05). Gram-negative bacilli, including *K. pneumoniae* and *A. baumannii* complex, *M. tuberculosis*, influenza A/B and SARS-CoV2 were detected in both CAP and HAP cases with no statistical difference.

### Seasonal variation of pathogens

**Figure 3.**
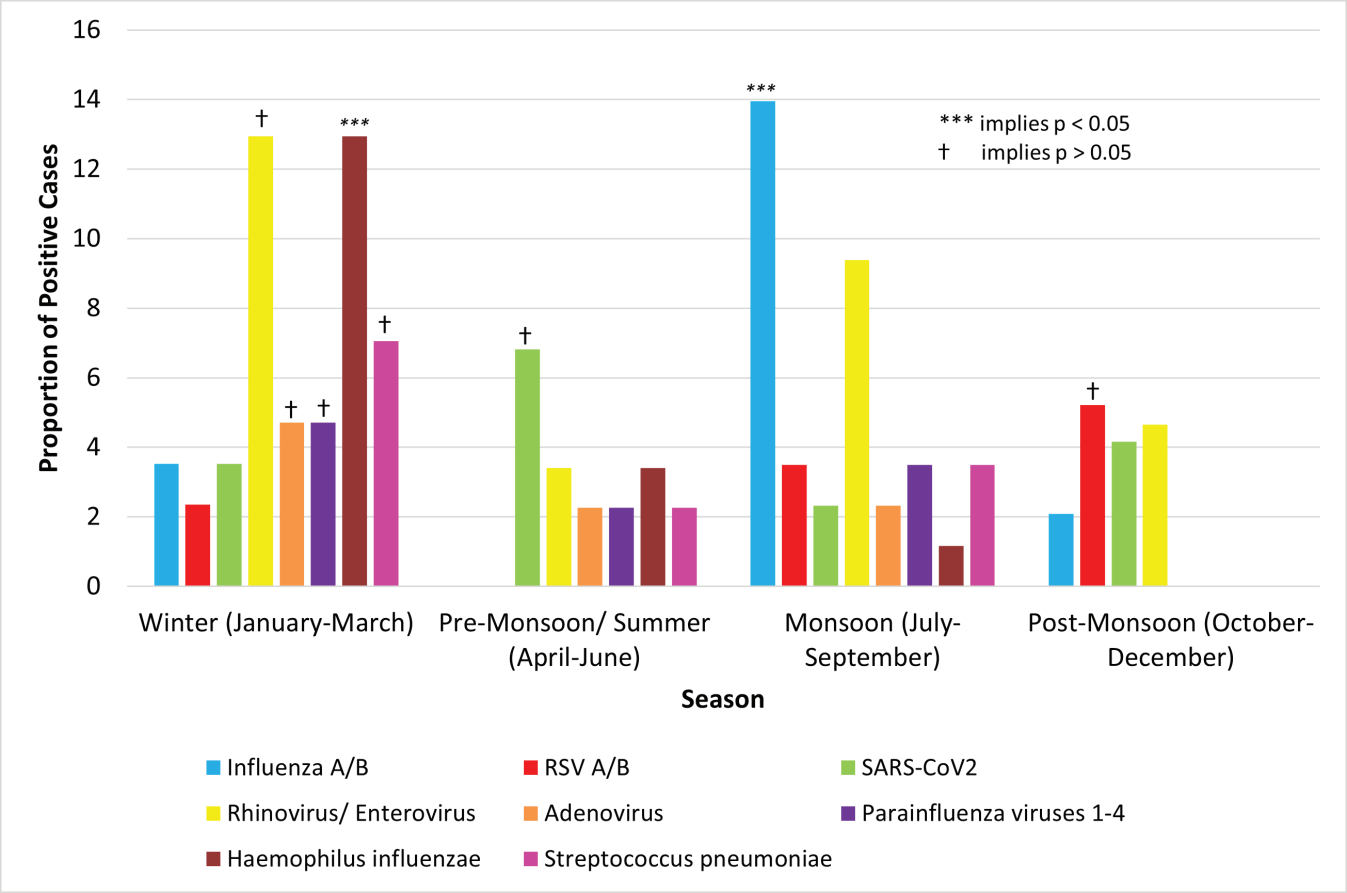
Seasonal variation of pathogens.

Significant seasonal variation (*p* < 0.001) was seen for influenza A/B and *H. influenzae* (Figure 3). The highest frequency of influenza A/B was during the monsoon (12 cases, 14.0%). The highest frequency of *H. influenzae* was in winter (11 cases, 12.9%).

Rhinovirus/enterovirus (11 cases, 12.9%), adenovirus (4, 4.7%), parainfluenza viruses 1–4 (4, 4.7%) and *S. pneumoniae* (6, 7.1%) had the highest incidence in winter. SARS-CoV2 was most frequent in the summer (6, 6.8%). RSV (5, 5.2%) had the highest incidence in post-monsoon. None of these showed significant seasonality (*p* > 0.05).

### Risk factors for pneumonia

Bone marrow transplant (BMT) patients had a 22 times increased risk of pneumonia. Haematological malignancies had an 11 times higher risk of pneumonia compared to patients with solid organ malignancies. Neutropenia (ANC <500/mcL) increased the risk of pneumonia by 9 times, thoracic malignancies by 7 times, chronic obstructive pulmonary disease (COPD) by 7 times, renal dysfunction by 5 times, chemotherapy by 4 times, radiotherapy by 3 times, immunotherapy by 3 times, diabetes by 2 times and smoking by 2 times.

A multivariable logistic regression model was constructed [18]. Adjusting for other variables in the model, COPD increased the risk of pneumonia 6 times, renal dysfunction 3 times, radiotherapy 3 times and diabetes 2 times (Table 3).

A sub-analysis in the pneumonia cohort showed that the distribution of solid organ malignancies, hematological malignancies and comorbidities in CAP and HAP was similar. Chemotherapy and radiotherapy were both more common in cases of CAP (*p* < 0.05). Surgery for cancer was much higher in cases of HAP (57.9%, *p* < 0.001).

### All-cause mortality in patients with pneumonia

Table 4 depicts the risk factors for all-cause mortality in patients with pneumonia. Patients with CAP had twice the risk of death from pneumonia compared to patients with HAP. Furthermore, patients with hematological malignancies had twice the risk of death compared to solid organ malignancies. Chemotherapy, renal dysfunction, neutropenia and immunotherapy increased the risk of death 2-fold. Surgical patients had a 66% lower risk.

In the multivariable Cox regression model, neutropenia increased the risk of pneumonia two-fold, adjusting for other predictors.

### Length of hospital stay in patients with pneumonia

Patients with pneumonia who had a BMT spent 18 days more in the hospital. Having HAP increased the length of hospital stay by 9 days, hematological malignancies by 5 days, co-infections by 5 days and diabetes by 3 days. Patients with pneumonia having a head and neck malignancy had a shorter length of hospital stay by 5 days and those post-radiotherapy by 4 days. A multivariable linear regression model was constructed, including variables with *p*-values ≤ 0.10. Adjusting for other variables, BMT increased hospital stay by 15 days, HAP by 10 days (compared to CAP), co-infection by 6 days and hypertension by 3 days (Table 5).

### Length of ICU stay in patients with pneumonia

BMT increased the length of ICU stay the most (9 days). HAP caused a mean of 4 days longer length of ICU stay than CAP. Co-infections increased the length of ICU stay by 5 days, hypertension by 3 days and diabetes by 3 days. On the other hand, patients with a head and neck malignancy had a 3-day shorter length of ICU stay (Table 6). Adjusting for other variables in the multivariable model, BMT increased the length of ICU stay by 8 days, co-infection by 6 days HAP 5 days (compared to CAP) and hypertension 3 days.

## Discussion

We conducted a 1-year prospective cohort study to investigate pneumonia in adult patients who were admitted to the oncology ICU. The incidence of pneumonia in this group was 15.6%, with the majority (51.8%) being community-acquired. The observed VAP rate (11.0 per 1000 ventilator days) was significantly higher than that reported in a multicentric study from India (6.4) [19]. This highlights a substantially higher VAP rate among cancer patients.

Patients with pneumonia had worse outcomes overall, in terms of 90-day all-cause mortality, mean length of hospital stay, ICU stay and mechanical ventilation. HAP (48% cases) increased the length of hospital and ICU stays. This emphasises the role that healthcare exposure plays in the burden of pneumonia.

Our microbiological positivity rate was 73% which is higher than similar studies done in oncology ICUs [7]. We used syndromic CB-NAATs along with conventional tests, resulting in higher sensitivity, in addition to rapid, multiplexed testing [20]. Our etiological findings allow for evidence-based antimicrobial and diagnostic stewardship in future cases of pneumonia in similar populations. Despite the numerous tests employed for pathogen identification, differentiating causal agents from colonisers can be challenging, particularly when less invasive samples (nasopharyngeal swabs, sputum) are used [21, 22].

*Klebsiella pneumoniae* was the commonest etiology in our study, followed by *P. aeruginosa*, *A. baumannii* complex and *S. aureus*. Globally, *S. pneumoniae* was the commonest cause of pneumonia, followed by *Mycoplasma*, *S. aureus*, *Chalmydia* and *H. influenzae*. *Klebsiella pneumoniae* was the eighth most common cause of pneumonia, *P. aeruginosa* tenth and *A. baumannii* twelfth [5]. In contrast, the ICMR antimicrobial resistance surveillance network reported that gram-negative bacilli constituted 84% of all lower respiratory isolates, of which 23% were *K. pneumoniae*, and *S. pneumoniae* constituted only 0.5% [23]. Notably, microscopy and culture show low diagnostic accuracy for *S. pneumoniae* due to its fastidious nature, contributing to the low apparent prevalence of the organism [24]. Immunodiagnostics and nucleic acid tests are required for increased sensitivity and specificity of identification [24].

Furthermore, we saw significant positivity of rhinovirus/enterovirus (27 cases), which is an uncommon cause of pneumonia in the general population [25], but may require care in the oncology setting. Furthermore, 19 cases. (70.4%) with rhinovirus/ enterovirus were co-infections.

Influenza A/B peaked during monsoon (July–September), similar to findings of other studies in latitudes below the tropic of cancer [26] and the CDC data [27]. This highlights the need for seasonal influenza vaccine roll-out conforming to the peak for our cohort. The peak of *H. influenzae* occurred in winter (January–March). This was consistent with European data for 2018, [28] but differed from CDC findings (bimodal) [29].

Diabetes, COPD and renal dysfunction increased the risk of pneumonia in multivariable analysis. This is in-line with the recommendations of the Advisory Committee on Immunisation Practices, which identifies these individuals as high risk for pneumococcal pneumonia [30]. Therefore, individuals with these comorbidities are at high risk and need vaccination. Radiotherapy also increased the risk of pneumonia, resulting from the immunosuppression it elicits. Similar findings have been previously reported [6]. On the other hand, patients who had undergone surgery had a 90% lower risk of developing pneumonia. The majority of the surgical patients (99.2%) underwent elective surgeries, which are offered to patients with a better baseline clinical status. This might contribute to the apparent decreased risk of pneumonia.

We observed that patients with CAP had twice the mortality rate than those with NV-HAP. This contradicts the understanding that HAP causes higher mortality [31]. Some community-acquired pathogens are highly virulent, causing rapid clinical deterioration without appropriate treatment. Pneumonia care in CAP depends on the patient reporting to the hospital, resulting in potential treatment delay. Furthermore, cancer patients have frequent healthcare exposures, which are not considered in the present ATS/IDSA definition for HAP [14]. Seventy percent of CAP patients had recent healthcare exposure, including 28% who were hospitalised, in the 30 days before the date of the event. An association between this exposure and the pneumonia episode cannot be eliminated.

Furthermore, gram-negatives such as *K. pneumoniae* and *A. baumannii*, usually considered hospital-acquired pathogens, were detected with no difference in distribution in CAP and HAP [14]. Similarly, rhinovirus/enterovirus, influenza A/B and SARS-CoV2, considered community-acquired pathogens, were detected in both CAP and HAP [32]. These findings highlight the inadequacy of the ATS/IDSA classification criteria in cohorts requiring frequent healthcare visits and the need for criteria specific to them.

Past studies have shown that hypertension increases the risk of pneumonia and causes worse outcomes in terms of mortality and the need for ICU care in patients with pneumonia [33, 34]. While we did not identify hypertension as a risk factor for pneumonia, we saw that hypertension increased lengths of both hospital and ICU stay by 3 days. HAP, BMT and co-infections also increased the length of hospital and ICU stays. Neutropenia increased the risk of mortality. We considered a comprehensive panel of patient characteristics as predictors for pneumonia and its outcomes in patients with cancer. Our findings would allow for patient stratification into risk groups for developing pneumonia and having adverse outcomes.

This study re-emphasises that pneumonia in cancer patients can be devastating, making prevention imperative. Vaccines for influenza A/B, *S. pneumoniae*, *H. influenzae* type b and SARS-CoV2, and RSV are recommended for immunocompromised adults [35, 36]. These pathogens were detected in 55 cases. Vaccination programs for adults, particularly in LMICs, are inadequate [37]. COVID-19 vaccination records were available for 21% patients in this study. Vaccination against *S. pneumoniae* and influenza vaccination was recorded in only 8 patients, and Hib conjugate vaccine in none. This suggests both a failure of vaccine uptake and documentation in a truly vulnerable population. We were, therefore, unable to enumerate the impact of vaccines in preventing pneumonia, and the incidence of vaccine breakthrough infections, both of which would further strengthen the evidence base for pneumonia in cancer patients.

Patients with risk factors for pneumonia should be monitored carefully to ensure early management in case infection develops. Where possible, patients with pneumonia, particularly those caused by *M. tuberculosis*, influenza and SARS-CoV2, should be in a negative pressure isolation room [38]. Hospital policies should ensure strict compliance with hand hygiene and respiratory etiquette (including face masks) guidelines among staff, patients and their relatives to prevent transmission of pathogens.

This study had limitations. The majority of the data were collected from the hospital management system, which may be incomplete. Smoking history was recorded based on self-reporting. Patients might not be forthcoming with this information due to fear of losing insurance cover or stigma [39], leading to missing observations (45% patients). This caused non-uniformity, which might induce bias in univariate analysis (OR= 1.7, 95% CI: 1.1–2.7). This variable had to be excluded from multivariable regression. Splenectomy [40] and proton pump inhibitor use [41], which have been shown to increase the risk of pneumonia, were not assessed in this study. Neither severity of malignancy nor staging was considered in this analysis, and therefore, presents as a limitation. Finally, we were unable to assess the effect of antibiotic prescription for pneumonia on mortality.

Despite its limitations, this study adds fundamentally to the evidence base of pneumonia in patients with cancer. Some findings can be extrapolated to other vulnerable patient groups, including the immunocompromised and the elderly. These patient groups are similar to adults with cancer in that they have weakened immune systems and are frequently in healthcare settings [42]. This implies that pneumonia etiology in these groups may be similar to patients with cancer, with gram-negatives forming the overwhelming majority, and higher susceptibility to opportunists such as *Aspergillus*, *P. jirovecii* and CMV. Furthermore, classification of pneumonia in the immunocompromised and elderly would be similarly complicated by frequent exposure to healthcare settings.

Our study establishes the criticality of pneumonia in patients with cancer by highlighting it as an important cause of hospital stay, ICU admission, mechanical ventilation and death. Our findings pave the way for the development of future infection control strategies, as well as research directed to prevent and treat pneumonia in patients with cancer. This lays the foundation for systematic surveillance of pneumonia, both CAP and HAP, in patients with cancer.

## Conclusion

The data gaps and limitations pave the way for future implementation research:

1. Expansion of similar surveillance for pneumonia in other oncology hospitals in LMICs is important to develop a strong evidence base in this patient group. An elaborate database is critical to the refinement of pneumonia stratification guidelines for CAP and HAP for patient cohorts with frequent healthcare exposures.
2. The surveillance of pneumonia in adult patients with cancer should be expanded to all in-patients. This would allow for a larger sample size and allow for the assessment of risk factors for severe pneumonia as well as those for ICU admission in pneumonia patients.
3. The findings of the present study can be used for the development of risk stratification models as well as clinical decision systems using machine learning for pneumonia and adverse clinical outcomes associated with it.
4. Streamlining documentation of vaccine uptake is necessary for a successful vaccination campaign. These data would allow for correlation studies between vaccine uptake and pneumonia, and the identification of vaccine breakthrough cases.
5. Mechanistic studies to explore the correlation of predictors to pneumonia are required.

## Conflict of interests

The authors declare that they have no competing interests.

## Institutional review statement

This was approved by the Institutional Ethics Committee of Tata Medical Center Kolkata (ID: 2022/TMC/252/1RB23). This was an observational study and did not interfere with patient care. The need for informed consent was waived by the Institutional Ethics Committee.

## Funding

Not applicable.

## Author contributions

The research question was articulated by S Bhattacharya, SD Bhattacharya and S Malik. The study protocol was developed by S Mukherjee, S Bhattacharya, SD Bhattacharya and S Malik. Cases were identified by S Bhattacharya, S Mukherjee, PS Ghosh, S Bagchi, G Goel, S Sen and S Malik. Data collection and database management was done by S Malik. The statistical analysis was done by S Malik, with guidance from SD Bhattacharya and DG Adhya. The manuscript was prepared by S Malik and SD Bhattacharya, and reviewed and edited by S Bhattacharya, S Mukherjee, PS Ghosh, S Bagchi, G Goel, S Sen, S Chatterji and DG Adhya.

## Figures and Tables

**Table 1.** Demographics and baseline clinical characteristics.

Characteristic	Patients with pneumonia (*n* = 355)	Patients without pneumonia (*n* = 356)	All patients (*n* = 711)
Demographics			
Age	64 (54–71)	60 (52–67)	62 (53-69)
Females	136 (38.3)	153 (43)	289 (40.6)
BMI	23.4 (20.8–26.8)	23.9 (21.1–26.7)	23.6 (21.0–26.7)
Malignancy type			
Solid organ tumors	258 (72.7)	344 (98.8)	602 (85.7)
Hematological malignancy	97 (27.3)	12 (1.2)	109 (14.3)
Type of admission			
Medical	279 (78.6)	63 (17.7)	342 (48.1)
Post-Surgical	76 (21.4)	293 (82.3)	369 (51.9)

Binary variables were described using frequency and proportions. Continuous variables (age and BMI) were describes using median and inter-quartile range

**Table 2.** Clinical outcomes in patients with and without pneumonia.

Outcome Measure	Patients with pneumonia (*n* = 355, 95% CI)	Patients without pneumonia (*n* = 356, 95% CI)	Hazard ratio (95% CI)	*p*-value
30-day all-cause mortality rate (per 1,000 person-days)	14.6 (12.3–17.5)	1.2 (0.7–2.0)	12.1 (6.7–21.9)	<0.001
60-day all-cause mortality rate (per 1,000 person-days)	9.1 (7.7–10.7)	0.8 (0.5–1.3)	10.4 (6.2–17.5)	<0.001
90-day all-cause mortality rate (per 1,000 person-days)	6.4 (5.4–7.6)	0.6 (0.4–1.0)	9.0 (5.6–14.5)	<0.001
Length of Hospital Stay (days)	18.6 (18.5–18.7)	12.2 (12.1–12.4)	-	<0.001
Length of ICU Stay (days)	10.9 (10.8–11.0)	3.8 (3.7–3.9)	-	<0.001

**Table 3.** Risk factors for pneumonia in adults in the ICU.

	Patients with pneumonia (*n* = 355)	Patients without pneumonia (*n* = 356)	Univariate analysis		Multivariable analysis	
			Odds ratio (95% CI)	*p*-value	Odds ratio (95% CI)	*p*-value
Malignancy type						
Haematological malignancy	97 (27.3)	12 (1.2)	10.8 (5.7–22.0)	<0.001	1.8 (0.8–3.8)	0.16
Solid organ malignancy	258 (72.7)	344 (98.8)	0.1 (0.0–0.2)	<0.001	−	−
Interventions						
Bone-marrow transplantation	15 (4.2)	0 (0.0)	22.0 (3.7–inf)	<0.001	3.4 (0.2–62.3)	0.41
Chemotherapy	237 (66.8)	118 (33.1)	4.1 (3.0–5.5)	<0.001	1.5 (1.0–2.2)	0.05
Immunotherapy	31 (8.8)	10 (2.8)	3.3 (1.6–6.9)	0.001	1.8 (0.8–4.2)	0.18
Radiotherapy	103 (29)	41 (11.5)	3.1 (2.1–4.7)	<0.001	2.6 (1.6–4.3)	<0.001
Surgery	152 (42.8)	325 (91.3)	0.1 (0.0–0.1)	<0.001	0.1 (0.1–0.2)	<0.001
- Chest	16 (10.5)	20 (6.2)	0.8 (0.4–1.6)	0.50	−	−
- Head and neck	48 (31.6)	90 (27.8)	0.5 (0.3–0.7)	0.004	−	−
- Tracheostomy related to surgery for cancer	29 (19.1)	57 (17.6)	0.5 (0.3–0.7)	0.002	−	−
- Abdominal	62 (40.8)	134 (41.4)	0.4 (0.2–0.5)	<0.001	−	−
- Genitourinary	34 (22.4)	81 (24.9)	0.4 (0.2–0.6)	<0.001	−	−
Co-morbidities						
Significant neutropenia (<500/mcL)	35 (10.0)	4 (1.2)	9.1 (3.2–25.9)	<0.001	1.6 (0.5–4.9)	0.39
Chronic Obstructive Pulmonary Disease (COPD)	20 (5.6)	3 (0.8)	7.0 (2.1–23.9)	0.002	5.9 (1.7–20.7)	0.006
Renal dysfunction (AKI or CKD)	30 (8.5)	6 (1.7)	5.4 (2.2–13.1)	<0.001	3.1 (1.1–8.2)	0.03
Diabetes	158 (44.5)	112 (31.5)	1.8 (1.3–2.4)	<0.001	1.6 (1.0–2.3)	0.02
Hypertension	209 (58.9)	186 (52.2)	1.3 (1.0–1.8)	0.08	1.1 (0.7–1.6)	0.65
Smoking	55 (28.5)	38 (19.1)	1.7 (1.1–2.7)	0.03	−	−
Asthma	7 (2.0)	7 (2.0)	1.0	−	−	−
Underweight (BMI < 16.5 kg/m^2^)	5 (1.4)	13 (3.7)	0.9 (0.5–1.6)	0.74	−	−
Obesity (BMI ≥ 30 kg/m^2^)	24 (6.8)	38 (10.7)	0.9 (0.7–1.3)	0.62	−	−

Risk factors for pneumonia were determined using logistic regression

**Table 4.** Risk factors for all-cause mortality by day 90 in pneumonia in adults with cancer.

	Univariate analysis		Multivariable analysis	
	Hazard ratio (95% CI)	*p*-value	Hazard ratio (95% CI)	*p*-value
Pneumonia				
Community-acquired pneumonia	1.6 (1.1–2.3)	0.007	1.1 (0.7–1.5)	0.77
Hospital-acquired pneumonia	0.6 (0.4–0.9)	0.007	–	–
Malignancy type				
Hematological malignancy	1.8 (1.3–2.6)	0.001	1.1 (0.7-1.6)	0.77
Solid organ malignancy	0.5 (0.4–0.8)	0.001	–	–
Interventions				
Chemotherapy	1.8 (1.2–2.6)	0.004	1.1 (0.7-1.7)	0.80
Immunotherapy	1.8 (1.1–2.9)	0.02	1.5 (0.9-2.6)	0.13
Bone-marrow transplantation	1.5 (0.7–3.1)	0.26	–	–
Radiotherapy	1.2 (0.9–1.8)	0.25	–	–
Surgery	0.3 (0.2–0.5)	<0.001	0.4 (0.3-0.7)	0.001
Co-morbidities				
Significant neutropenia (<500/mcL)	2.3 (1.4–3.6)	<0.001	1.6 (1.0-2.6)	0.04
Renal dysfunction (AKI or CKD)	1.7 (1.0–2.8)	0.04	1.3 (0.8-2.2)	0.32
Chronic Obstructive Pulmonary Disease (COPD)	1.6 (0.8–3.0)	0.17	–	–
Smoking	1.3 (0.8–2.0)	0.34	–	–
Hypertension	1.1 (0.8–1.5)	0.75	–	–
Diabetes	1.0 (0.7–1.3)	0.68	–	–
Underweight (BMI < 18.5 kg/m^2^)	1.0 (0.5–3.0)	0.99	–	–
Obesity or Overweight (BMI ≥ 25 kg/m^2^)	0.7 (0.5–1.1)	0.10	–	–
Hospitalization				
Days of Hospital Stay	1.0 (0.9–1.0)	0.20	–	–
Days of ICU Stay	1.0 (0.9–1.0)	0.10	–	–
Days of Mechanical Ventilation	1.0 (0.9–1.0)	0.13	–	–
Number of pathogens detected				
0	Reference	Reference	Reference	Reference
1	0.9 (0.6–1.4)	0.61	0.8 (0.5–1.3)	0.36
≥ 2	1.5 (1.0–2.3)	0.07	1.2 (0.8–1.9)	0.35

Risk factors for mortality in patients with pneumonia were determined using the Cox proportional hazard model

**Table 5.** Determinants of length of hospital stay in patients with pneumonia.

	Univariate analysis		Multivariable analysis	
	Additional days of hospital stay (95% CI)	*p*-value	Additional days of hospital stay (95% CI)	*p*-value
Pneumonia				
Hospital-acquired pneumonia	9.3 (6.6–12.0)	<0.001	9.6 (7.0–12.2)	<0.001
Community-acquired pneumonia	Reference	Reference	–	–
Malignancy type				
Hematological malignancy	4.9 (1.7–8.1)	<0.001	2.1 (-1.1-5.4)	0.19
Solid organ malignancy	Reference	Reference	–	–
Interventions				
Bone-marrow transplantation	18.3 (11.4–25.2)	<0.001	15.4 (8.6–22.1)	<0.001
Chemotherapy	0.4 (-2.8-3.4)	0.82	–	–
Surgery	−0.2 (-3.1-2.7)	0.88	–	–
Immunotherapy	−0.7 (-5.8-4.4)	0.78	–	–
Radiotherapy	−4.2 (-(7.4-1.1))	0.009	-2.1 (-5.1-0.9)	0.16
Co-morbidities				
Diabetes	3.1 (0.2-6.0)	0.04	1.0 (-1.8-3.3)	0.48
Hypertension	2.5 (-0.4-5. 5)	0.09	3.0 (0.3–5.7)	0.03
Significant neutropenia (<500/mcL)	0.7 (-4.1-5.6)	0.76	–	–
Renal dysfunction (AKI or CKD)	0.1 (-5.1-5.3)	0.98	–	–
Underweight (BMI < 18.5 kg/m^2^)	−0.4 (-6.0-5.7)	0.96	–	–
Obesity or Overweight (BMI ≥ 25 kg/m^2^)	−0.6 (-4.2-2.9)	0.73	–	–
Smoking	−3.6 (-7.6-0.4)	0.81	–	–
Chronic obstructive pulmonary disease (COPD)	−4.2 (-10.4-2.1)	0.19	–	–
Number of pathogens detected				
0	Reference	Reference	Reference	Reference
1	1.5 (-2.1-5.0)	0.42	1.6 (-1.6-4.8)	0.32
≥ 2	5.0 (1.3–8.6)	0.008	6.2 (2.8–9.5)	<0.001

Determinants of length of hospital stay in patients with pneumonia were identified using linear regression. Additional days of stay were calculated using regression coefficients. Negative number of days imply a decrease in length of stay

**Table 6.** Determinants of length of ICU stay in patients with pneumonia.

	Univariate analysis		Multivariable analysis	
	Additional days of ICU stay (95% CI)	*p*-value	Additional days of ICU stay (95% CI)	*p*-value
Pneumonia				
Hospital-acquired pneumonia	4.1 (2.1–6.1)	<0.001	4.5 (2.6–6.5)	<0.001
Community-acquired pneumonia	Reference	Reference	–	–
Malignancy type				
Hematological malignancy	1.9 (-0.5-4.2)	0.11	–	–
Solid organ malignancy	Reference	Reference	–	–
Interventions				
Bone-marrow transplantation	8.7 (3.6-13.8)	0.001	8.2 (3.4–13.0)	0.001
Immunotherapy	0.5 (-3.2-4.1)	0.80	–	–
Chemotherapy	-0.4 (-2.6-1.8)	0.71	–	–
Surgery	-0.8 (-2.9-1.3)	0.46	–	–
Radiotherapy	-1.9 (-4.2-0.4)	0.10	-1.5 (-3.6-0.7)	0.19
Co-morbidities				
Hypertension	2.8 (0.7–4.9)	0.008	2.9 (0.9–5.0)	0.006
Diabetes	2.7 (0.6–4.8)	0.01	1.0 (-1.1-3.0)	0.36
Significant neutropenia (<500/mcL)	0.5 (-3.0-3.9)	0.79	–	–
Renal dysfunction (AKI or CKD)	-0.1 (-3.9-3.6)	0.94	–	–
Underweight (BMI < 18.5 kg/m^2^)	-0.1 (-4.3-4.1)	0.97	–	–
Obesity or Overweight (BMI ≥ 25 kg/m^2^)	-0.3 (-2.9-2.3)	0.81	–	–
Chronic obstructive pulmonary disease (COPD)	-1.7 (-6.2-2.9)	0.46	–	–
Smoking	-2.0 (-5.1-1.0)	0.19	–	–
Number of pathogens detected				
0	Reference	Reference	Reference	Reference
1	1.4 (-1.1-4.0)	0.27	1.6 (-0.8-4.0)	0.19
≥ 2	5.4 (2.8–8.0)	<0.001	6.1 (3.6–8.5)	<0.001

Determinants of length of ICU stay in patients with pneumonia were identified using linear regression. Additional days of stay were calculated using regression coefficients. Negative number of days imply a decrease in length of stay

## Supplementary Tables

**Supplementary Table S1.** Pneumonia case definition based on the National Health Safety Network [1].

Signs/ Symptoms	AND	Imaging test evidence
At least one of the following: • Fever (> 38.0°C or > 100.4°F) • Leukopenia (≤ 4,000 WBC/mm^3^) or leukocytosis (≥ 12,000 WBC/mm^3^) • For adults ≥ 70 years old, altered mental status with no other recognized cause AND At least two of the following: • New onset of purulent sputum or change in character of sputum, or increased respiratory secretions, or increased suctioning requirements • New onset or worsening cough, or dyspnea, or tachypnea • Rales or bronchial breath sounds on auscultation • Worsening gas exchange (for example: O_2_ desaturations (for example: PaO_2_/FiO_2_ ≤ 240), increased oxygen requirements, or increased ventilator demand)		Two or more serial chest imaging test results with at least one of the following: New and persistent OR Progressive and persistent Infiltrate Consolidation Cavitation

**Supplementary Table S2.** Presence of radiological and clinical features.

Feature	Frequency (%)
Clinical features (*n* = 276)	
Fever	109 (39.5)
New onset of purulent sputum or change in character of sputum, or increased respiratory secretions, or increased suctioning requirements	42 (15.2)
New onset or worsening cough or dyspnea or tachypnea	166 (60.1)
Rales or bronchial breath sounds	26 (9.4)
Worsening gas exchange	113 (40.9)
Altered mental status (aged > 70 years)	9 (3.3)
Radiological features (*n* = 355)	
Infiltration	70 (19.7)
Consolidation	288 (81.1)
Cavitation	8 (2.3)
